# Vitamin D Levels Decline with Rising Number of Cardiometabolic Risk Factors in Healthy Adults: Association with Adipokines, Inflammation, Oxidative Stress and Advanced Glycation Markers

**DOI:** 10.1371/journal.pone.0131753

**Published:** 2015-06-29

**Authors:** Zora Krivošíková, Martin Gajdoš, Katarína Šebeková

**Affiliations:** 1 Department of Clinical and Experimental Pharmacotherapy, Medical Faculty, Slovak Medical University, Bratislava, Slovakia; 2 Institute of Molecular BioMedicine, Medical Faculty, Comenius University, Bratislava, Slovakia; Queen's University Belfast, UNITED KINGDOM

## Abstract

**Introduction:**

Hypovitaminosis D associates with obesity, insulin resistance, hypertension, and dyslipoproteinemia. We asked whether the presence of multiple cardiometabolic risk factors, and which particular combination, exerts additive negative effects on 25(OH)D_3_ levels; and whether 25(OH)D_3_ levels associate with markers of inflammation and oxidative stress.

**Subjects and Methods:**

In non-diabetic medication-free adults central obesity (waist-to-height ratio > 0.5); elevated blood pressure (systolic BP≥130 mm Hg and/or diastolic BP ≥85 mm Hg); increased atherogenic risk (log(TAG/HDL) ≥ 0.11); and insulin resistance (QUICKI < 0.322) were considered as cardiometabolic risk factors. 25(OH)D_3_ status was classified as deficiency (25(OH)D_3_ ≤20 ng/ml); insufficiency (levels between 20-to-30 ng/ml), or as satisfactory (>30 ng/ml). Plasma adipokines, inflammatory and oxidative stress markers, advanced glycation end-products, and their soluble receptor were determined.

**Results:**

162 subjects were cardiometabolic risk factors-free, 162 presented increased (i.e. 1 or 2), and 87 high number (i.e. 3 or 4) of cardiometabolic risk factors. Mean 25(OH)D_3_ decreased with rising number of manifested risk factors (36 ± 14 ng/ml, 33 ± 14 ng/ml, and 31 ± 15 ng/ml, respectively; p_ANOVA_: 0.010), while prevalence of hypovitaminosis D did not differ significantly. Elevated blood pressure and insulin resistance appeared as significant determinants of hypovitaminosis D. Subjects presenting these risk factors concurrently displayed the lowest 25(OH)D_3_ levels (29 ± 15 ng/ml). Plasma adipokines, inflammatory and oxidative stress markers, advanced glycation end-products, and their soluble receptor generally differed significantly between the groups, but only advanced oxidation protein products and advanced glycation end-products associated fluorescence of plasma showed significant independent association with 25(OH)D_3_ levels.

**Conclusion:**

In apparently healthy adults increasing number of cardiometabolic risk factors associates with poorer 25(OH)D_3_ status, while the association between 25(OH)D_3_ status and inflammatory or oxidative stress markers remains equivocal.

## Introduction

Steroid hormone vitamin D is produced in dermis from 7-dehydrocholesterol through exposure to UV-B irradiation. Provitamin D_3_ quickly undergoes rearrangements to form stable vitamin D_3_, and enters circulation after binding to vitamin D binding protein (DBP). It is hydroxylated to 25(OH)D_3_ in the liver and thereafter to its active form 1,25(OH)_2_D_3_ by 1, α-hydroxylase in the kidneys. Moreover, a wide variety of extrarenal cells expressing vitamin D nuclear receptor—VDR (i.e. adipocytes, cells of the immune system, colon, pancreas, skin and the vasculature) synthesize 1,25(OH)_2_D_3_. This local production of vitamin D is responsible for extraskeletal modulation of various physiological processes: complex of vitamin D, VDR, and other transcription factors (such as retinoid X receptor); and binds to vitamin D response elements of as many as 2000 genes to regulate their expression directly or in directly [1]. Hence hypovitaminosis D extends its negative effects beyond calcium homeostatsis and skeletal health playing a pathophysiological role in different noncommunicable diseases.

Vitamin D deficiency may contribute to manifestation of different cardiometabolic risk factors via distinct biological pathways. Vitamin D may modulate blood pressure (BP) by suppression of the renin–angiotensin–aldosterone system, direct effects on vascular cells, renoprotective effects, and those on calcium metabolism, including prevention of secondary hyperparathyroidism [2–4]. Link between vitamin D and glucose homeostasis is complex. Pancreatic β-cells express vitamin D receptors and 1, α-hydroxylase enzymes, and vitamin D response element is present in insulin gene promoter region [5, 6]. Thus, vitamin D affects insulin synthesis, release, β-cell function, as well as insulin sensitivity [7–9]. Moreover, polymorphisms in vitamin DBP, VDR, and 1, α-hydroxylase genes might link vitamin D deficiency to insulin resistance (reviewed in [10]). Mitochondria possess both a functional renin-angiotensin system and vitamin D receptors, and their dysfunction has been implicated in the pathogenesis of hypertension and insulin resistance via enhanced generation of reactive oxygen species, induction of endoplasmic-reticulum stress, and inflammation [11]. Also adipocytes express vitamin D receptors and 1, α-hydroxylase enzymes. Vitamin D may affect body fat mass by inhibiting lipid accumulation and key adipogenic transcription factors during adipocyte differentiation [12]. It may modulate lipid profile indirectly through its effect on serum parathormone and/or on the calcium balance, or via modulation of insulin secretion and sensitivity [13–16].

Several clinical studies indicate an association between inadequate vitamin D status and single cardiometabolic risk factors such as obesity (central obesity in particular), hyperglycemia, insulin resistance, hypertension, dyslipoproteinaemia, and disbalance of immune function (reviewed in [1, 17–19]). In general population, obese subjects, patients with hypertension or impaired fasting glucose, low 25(OH)D_3_ levels associate with higher levels of systemic inflammation, glycoxidative, lipoxidative, and carbonyl stress markers [20, 21]. Vitamin D influences production of adipokines and the inflammatory response in adipose tissue [12]. It regulates genes encoding pro-inflammatory cytokines, downregulates Toll-like receptors expression [8, 9], and counteracts the inflammatory effects induced by cytokines [22, 23]. Thus, hypovitaminosis D (serum 25(OH)D_3_ levels <30 ng/ml are classified as insufficiency; those <20 ng/ml as deficiency) represents a global problem [24].

Owing to distinct pathomechanisms linking different cardiometabolic risk factors to vitamin D status, we hypothesized that presence of multiple cardiometabolic risk factors should be associated with lower 25(OH)D_3_ levels and a higher prevalence of hypovitaminosis D. We also asked which particular combination of cardiometabolic risk factors associates with poor 25(OH)D_3_ status. To this point, non-diabetic apparently healthy adults presenting increased (1 or 2) and high number (3 or 4) of cardiometabolic risk factors were compared with their cardiometabolic risk factors-free counterparts. We also studied the association of vitamin D_3_ status with non-standard cardiometabolic risk factors, such as adipokines, markers of microinflammation and oxidative stress, advanced glycation end products (AGEs), and their soluble receptor (sRAGE).

## Subjects and methods

This cross-sectional study was conducted in accordance to the principles of the Declaration of Helsinki. The study protocol was approved by the Ethics Committee of the Slovak Medical University in Bratislava. All subjects signed an informed consent to participate.

### Study population

Apparently healthy volunteers residing in Bratislava and surroundings, declaring that they do not suffer from and are not treated for any acute or chronic illness, and did not take any vitamin D supplements during last 6 months, were recruited. Elevated fasting plasma glucose (FPG ≥ 7 mmol/l), decreased renal function (eGFR ≤ 0.6 ml/s/1.73m^2^), pregnancy and lactation were exclusion criteria.

From among 452 recruited subjects aged 18-to-81 years who underwent blood sampling during winter period 41 were excluded: 11 in whom 25(OH)D_3_ levels were not determined for technical reasons, 17 presenting FPG ≥ 7 mmol/l, 2 with eGFR ≤ 0.6 ml/s/1.73m^2^, and 11 with incomplete data for unequivocal classification of cardiometabolic risk factors. Thus, data from 411 subjects was available for analysis.

### Procedures

Body weight, height, waist circumference, and BP were measured in the outpatients department in the morning hours. Blood pressure was measured at forearm in sitting position after 10 min. rest and the mean of last 2 measurements out of 3 taken was recorded. Self-reported smoking status was recorded (current smoker/non-smoker). Blood was taken from antecubital vein after overnight fasting.

### Research variables

Standard blood chemistry (glucose, lipid profile, creatinine, uric acid, albumin) was analyzed immediately (Vitros 250 analyzer, Johnson&Johnson, Rochester, NY, USA). Aliquoted plasma samples were stored at -80°C for special analyses. Commercial ELISA sets according to manufacturers’ instructions were used to determine high sensitive C-reactive protein (hsCRP, Immundiagnostik AG, Bensheim, Germany), interleukine-6 (Human IL-6, R&D Systems, Minneapolis, MN, USA), high sensitive transforming growth factor-α (Human TNF-α/TNFSF1A, R&D Systems, Minneapolis, MN, USA), adiponectin (Human Adiponectin, R&D Systems, Minneapolis, MN, USA), leptin (Human Leptin, R&D Systems, Minneapolis, MN, USA), resistin (Human Resistin, R&D Systems, Minneapolis, MN, USA), N^ε^-carboxymethyllysine (CML, Microcoat, Bernried, Germany) and soluble receptor for advanced glycation end products (sRAGE, R&D Systems, Minneapolis, MN, USA). Serum concentrations of 25-hydroxyvitamin D was analyzed by RIA method (25(OH)D_3_, Immuno Diagnostic system, Boldon, UK) and intact parathyroid hormone by IRMA method (PTH, Immunotech, Marseille, France). Advanced oxidation protein products (AOPPs) were determined after precipitation of plasma lipids [25, 26], and advanced glycation end products-associated fluorescence of plasma (AGE-Fl) according to Munch et al. [27].

Body mass index (BMI), waist-to-height ratio (index of central obesity, ICO), mean arterial pressure (MAP), pulse pressure (PP), quantitative insulin sensitivity check index (QUICKI) [28], atherogenic index of plasma (AIP, log(TAG/HDL)) [29], and glomerular filtration rate (eGFR) [30] were calculated. CML, AGE-Fl and AOPPs were corrected for plasma albumin.

### Classification of cardiometabolic risk factors

Presence of cardiometabolic risk factors was classified as follows: central obesity: ICO>0.5 [31]; elevated BP: systolic BP (SBP)≥130 mm Hg and/or diastolic BP (DBP)≥85 mm Hg; increased atherogenic risk: AIP≥0.11 [29]; insulin resistance: QUICKI<0.322. Subjects were classified into 3 groups: those not presenting cardiometabolic risk factors; subjects presenting increased (i.e. 1 or 2); and those presenting high number (i.e. 3 or 4) of risk factors.

### Vitamin D status classification

Vitamin D status was classified as deficiency if plasma vitamin D concentrations ≤20 ng/ml, as insufficiency if levels ranged between 20-to-30 ng/ml, while higher levels were considered as satisfactory.

### Statistical analysis

Normality of data distribution and equality of variances were tested (Kolmogorov-Smirnov and Levene’s test, respectively). Skewed data were logarithmically (ln) transformed for statistical evaluation, but for better understanding data are given as mean ± standard deviation (x ± SD); if not given differently. Two groups were compared using Student’s t-test, comparison between 3 groups was performed using one way analysis of variance (ANOVA) with post-hoc Scheffe’s test. Categorical data were compared by chi-square test. General linear model (GLM) was employed to study the impact of cardiometabolic risk factors on 25(OH)D_3_ status. Binary logistic regression analysis was used to provided evidence on whether the cardiometabolic risk factors, gender and renal function could be considered independent variables for prediction of satisfactory 25(OH)D_3_ status. In Model 1 number of cardiometabolic risk factors, gender, and ln eGFR were used as predictors; in Model 2 number of risk factors was replaced by categories (absence/presence) of elevated blood pressure, insulin resistance, central obesity and atherogenic index. Classification tree was employed to study the impact of independent variables on 25(OH)D_3_ levels. P <0.05 (2-sided) was considered significant. Statistical analyses were performed using program SPSS version 22 (SPSS Inc., Chicago, IL, USA).

## Results

### Cohort characteristics

Study participants were 36 ± 14 years of age, on average slightly overweight (average BMI: 25 ± 5 kg/m^2^) and not presenting central obesity (ICO: 0.50 ± 0.08), (Table 1). Except for mildly decreased mean eGFR they presented mean BP values, variables characterizing glucose homeostasis and lipid metabolism, concentrations of uric acid, albumin, and inflammatory markers (hsCRP, IL-6) within the reference range (Tables 1 and 2).

**Table 1:** Cohort characteristics: anthropometric data, blood pressure, standard blood chemistry variables and 25(OH)D_3_ levels.

		Number of cardiometabolic risk factors			
	All	0	1–2	3–4	p (ANOVA
N	411	162	162	87	
Gender (F/M) (n;%)	240/171(58%/42%)	129/33(80%/20%)	87/75(54%/46%)	24/63(28%/72%)	**0.001** ^**chi**^
Age (years)	*35*.*9 ± 14*.*1*	*30*.*0 ± 8*.*9*	*36*.*2 ± 13*. *9* ***	*46*.*1 ± 14*.*6* *** ^,^ ^*+++*^	***<0*.*001***
Weight (kg)	*74*.*4 ± 17*.*1*	*62*.*9 ± 9*.*7*	*76*.*8 ± 15*.*4* ***	*91*.*5 ± 15*.*1* *** ^,^ ^*+++*^	***<0*.*001***
Waist (cm)	*86*.*2 ± 14*.*6*	*75*.*0 ± 6*.*7*	*87*.*9 ± 11*.*3* ***	*104*.*1 ± 11*.*0* *** ^,^ ^*+++*^	***<0*.*001***
Height (cm)	*172*.*0 ± 9*.*7*	*170*.*1 ± 8*.*5*	*172*.*6 ± 10*.*9*	*174*.*1 ± 8*.*7* **	***0*.*004***
BMI (kg/m^2^)	*25*.*1 ± 4*.*8*	*21*.*6 ± 2*.*1*	*25*.*7 ± 4*.*1* ***	*30*.*2 ± 4*.*3* *** ^,^ ^*+++*^	***<0*.*001***
ICO	*0*.*50 ± 0*.*08*	*0*.*44 ± 0*.*03*	*0*.*51 ± 0*.*07* ***	*0*.*60 ± 0*.*07* *** ^,^ ^*+++*^	***<0*.*001***
SBP (mm Hg)	*124*.*4 ± 14*.*4*	*114*.*4 ± 7*.*5*	*127*.*2 ± 13*.*3* ***	*137*.*6 ± 13*.*1* *** ^,^ ^*+++*^	***<0*.*001***
DBP (mm Hg)	*76*.*7 ± 8*.*5*	*72*.*0 ± 6*.*5*	*78*.*0 ± 7*.*8* ***	*83*.*4 ± 7*.*6* *** ^,^ ^*+++*^	***<0*.*001***
MAP (mm Hg)	*92*.*6 ± 9*.*5*	*86*.*1 ± 5*.*7*	*94*.*4 ± 8*.*4* ***	*101*.*5 ± 8*.*1* *** ^,^ ^*+++*^	***<0*.*001***
PP (mm Hg)	*47*.*7 ± 11*.*1*	*42*.*5 ± 7*.*9*	*48*.*3 ± 11*.*3* ***	*49*.*3 ± 11*.*3* *** ^,^ ^*++*^	***<0*.*001***
FPG (mmol/l)	*5*.*2 ± 0*.*6*	*4*.*9 ± 0*.*4*	*5*.*1 ± 0*.*6* ***	*5*.*7 ± 0*.*7* *** ^,^ ^*+++*^	***<0*.*001***
FIns (μIU/ml)	*10*.*0 ± 8*.*3*	*6*.*9 ± 2*.*6*	*9*.*6 ± 7*.*8* **	*16*.*5 ± 11*.*6* *** ^,^ ^*+++*^	***<0*.*001***
QUICKI	0.351 ± 0.035	0.365 ± 0.025	0.354 ± 0.037**	0.320 ± 0.029*** ^,^ ^*+++*^	**<0.001**
Chol (mmol/l)	4.7 ± 0.9	4.4 ± 0.8	4.6 ± 0.9**	5.3 ± 0.8*** ^,^ ^*+++*^	**<0.001**
HDL-C (mmol/l)	*1*.*4 ± 0*.*4*	*1*.*5 ± 0*.*4*	*1*.*4 ± 0*.*4* ***	*1*.*1 ± 0*.*2* *** ^,^ ^*+++*^	***<0*.*001***
LDL-C (mmol/l)	2.7 ± 0.8	2.4 ± 0.7	2.7 ± 0.8**	3.2 ± 0.7*** ^,^ ^*+++*^	**<0.001**
VLDL-C (mmol/l)	*0*.*5 ± 0*.*3*	*0*.*4 ± 0*.*2*	*0*.*5 ± 0*.*3* ***	*0*.*9 ± 0*.*3* *** ^,^ ^*+++*^	***<0*.*001***
TAG (mmol/l)	*1*.*3 ± 0*.*9*	*0*.*8 ± 0*.*3*	*1*.*2 ± 0*.*7* ***	*2*.*2 ± 1*.*1* *** ^,^ ^*+++*^	***<0*.*001***
AIP	-0.10 ± 0.33	-0.30 ± 0.21	-0.10 ± 0.30***	0.28 ± 0.24*** ^,^ ^*+++*^	**<0.001**
Albumin (g/l)	47.8 ± 5.3	47.6 ± 5.3	48.2 ± 5.2	46.8 ± 5.6	0.26
Creatinine (μmol/l)	*85*.*8 ± 16*.*0*	*80*.*7 ± 13*.*0*	*87*.*7 ± 17*.*3* ***	*92*.*0 ± 15*.*9* ***	***0030***
eGFR (ml/s/1.73m^2^)	*1*.*29 ± 0*.*24*	*1*.*34 ± 0*.*21*	*1*.*28 ± 0*.*23* *	*1*.*23 ± 0*.*25* ***	***0*.*001***
Uric acid (mmol/l)	281 ± 75	251 ± 63	289 ± 73***	354 ± 63*** ^,^ ^*+++*^	**<0.001**
Vitamin D_3_ (ng/ml)	*33*.*4 ± 14*.*5*	*35*.*5 ± 14*.*2*	*32*.*6 ±14*.*3* *	*31*.*0 ± 15*.*1* *	***0*.*010***
PTH (pg/ml)	*43*.*1 ± 24*.*2*	*35*.*5 ± 19*.*5*	*45*.*1 ± 23*.*3* **	*53*.*5± 28*.*9* *** ^,^ ^*+*^	***<0*.*001***
Smoking (yes/no)	101/30 (25%/75%)	40/122 (25%/75%)	43/119(27%/73%)	18/69(21%/79%)	0.59^chi^

Central obesity: waist-to-height ration >0.5 [31]; elevated BP: systolic BP (SBP)≥130 mm Hg and/or diastolic BP (DBP)≥85 mm Hg; increased atherogenic risk: AIP≥0.11 [29]; and insulin resistance: QUICKI<0.322 were considered as factors indicated increased cardiometabolic risk. 0: no risk factor presented (n = 162); 1–2: subjects with 1 or 2 risk factors (n = 162); 3–4: subjects presenting 3 or 4 risk factors (n = 87); F: females; M: males; BMI: body mass index; ICO: index of central obesity; SBP: systolic blood pressure; DBP: diastolic blood pressure; MAP: mean arterial pressure; PP: pulse pressure; FPG: fasting plasma glucose; FIns: fasting plasma insulin; QUICKI: quantitative insulin sensitivity check index; CHOL: total cholesterol; HDL-C: high density lipoprotein cholesterol; LDL-C: low density lipoprotein cholesterol; VLDL-C: very low density lipoprotein cholesterol; TAG: triacylglycerols; AIP: atherogenic index of plasma; eGFR: estimated glomerular filtration rate; Vitamin D_3_: plasma 25(OH)D_3_; PTH: intact parathormone; chi: chi-square

*: p<0.05 vs. cardiometabolic risk factors free subjects

**: p<0.01 vs. cardiometabolic risk factors free subjects

***: p<0.001 vs. cardiometabolic risk factors free subjects

+: p<0.05 vs. subjects presenting 1 or 2 cardiometabolic risk factors

++: p<0.01 vs. subjects presenting 1 or 2 cardiometabolic risk factors

+++: p<0.001 vs. subjects presenting 1 or 2 cardiometabolic risk factors; *italics*: statistical evaluation performed on logarithmically transformed data

**Table 2:** Cohort characteristics: inflammatory and oxidative stress markers, adipokines and advanced glycation end products.

		Number of cardiometabolic risk factors			
	All	0	1–2	3–4	p (ANOVA)
N	411	162	162	87	
hsCRP (mg/l)	*2*.*3 ± 3*.*2*	*1*.*7 ± 2*.*9*	*2*.*5 ± 3*.*3* **	*3*.*3 ± 3*.*3* *** ^,^ ^*++*^	***<0*.*001***
IL-6 (pg/ml)	*2*.*9 ± 2*.*7*	*2*.*9 ± 3*.*0*	*3*.*0 ± 2*.*7*	*2*.*5 ± 1*.*9*	*0*.*36*
hsTNF-α (pg/ml)	*2*.*5 ± 3*.*3*	*2*.*2 ± 2*.*0*	*2*.*8 ± 3*.*5*	*2*.*5 ± 4*.*5*	*0*.*09*
Adiponectin (μg/ml)	*8*.*2 ± 4*.*8*	*9*.*6 ± 4*.*6*	*8*.*1 ± 4*.*9* ***	*5*.*6 ± 3*.*8* *** ^,^ ^*+++*^	***<0*.*001***
Leptin (ng/ml)	*15*.*9 ± 21*.*4*	*13*.*3 ± 10*.*5*	*14*.*7 ± 14*.*1*	*22*.*9 ± 39*.*2* *** ^,^ ^*++*^	***<0*.*001***
Resistin (ng/ml)	*10*.*7 ± 4*.*0*	*10*.*1 ± 3*.*5*	*10*.*6 ± 4*.*1*	*12*.*1 ± 4*.*4* *** ^,*+*^	***<0*.*001***
CML/Alb (μg/g)	*26*.*2 ± 6*.*7*	*27*.*4 ± 6*.*7*	*25*.*6 ± 6*.*3*	*24*.*0 ± 7*.*1* *	***0*.*014***
AGE-Fl/Alb (AU/g)	*6*.*3 ± 2*.*0*	*6*.*3 ± 2*.*2*	*6*.*0 ± 1*.*8*	*7*.*0 ± 2*.*2* ^*++*^	***0*.*007***
AOPP/Alb (μmol/g)	*1*.*6 ± 0*.*9*	*1*.*5 ± 0*.*9*	*1*.*7 ± 0*.*9*	*1*.*6 ± 0*.*8*	*0*.*06*
sRAGE (pg/ml)	*1263 ± 440*	*1320 ± 456*	*1265 ± 428*	*1073 ± 374* ** ^,^ ^*+*^	***0*.*001***

Central obesity: waist-to-height ration >0.5 [31]; elevated BP: systolic BP (SBP)≥130 mm Hg and/or diastolic BP (DBP)≥85 mm Hg; increased atherogenic risk: AIP≥0.11 [29]; and insulin resistance: QUICKI<0.322 were considered as factors indicated increased cardiometabolic risk. 0: no risk factor presented (n = 162); 1–2: subjects with 1 or 2 risk factors (n = 162); 3–4: subjects presenting 3 or 4 risk factors (n = 87); hsCRP: high sensitive C-reactive protein; IL-6: interleukine-6; hsTNF-α: high sensitive tumor necrosis factor-α; CML: N^ε^-(carboxymethyl)lysine; Alb: albumin; AGE-Fl: advanced glycation end products associated fluorescence of plasma; AU: arbitrary units; AOPP: advanced oxidation protein products; sRAGE: soluble receptor for advanced glycation end products

*: p<0.05 vs. cardiometabolic risk factors free subjects

**: p<0.01 vs. cardiometabolic risk factors free subjects

***: p<0.001 vs. cardiometabolic risk factors free subjects

+: p<0.05 vs. subjects presenting 1 or 2 cardiometabolic risk factors

++: p<0.01 vs. subjects presenting 1 or 2 cardiometabolic risk factors

+++: p<0.001 vs. subjects presenting 1 or 2 cardiometabolic risk factors; *italics*: statistical evaluation performed on logarithmically transformed data

### 25(OH)D_3_ status

**Fig 1:**
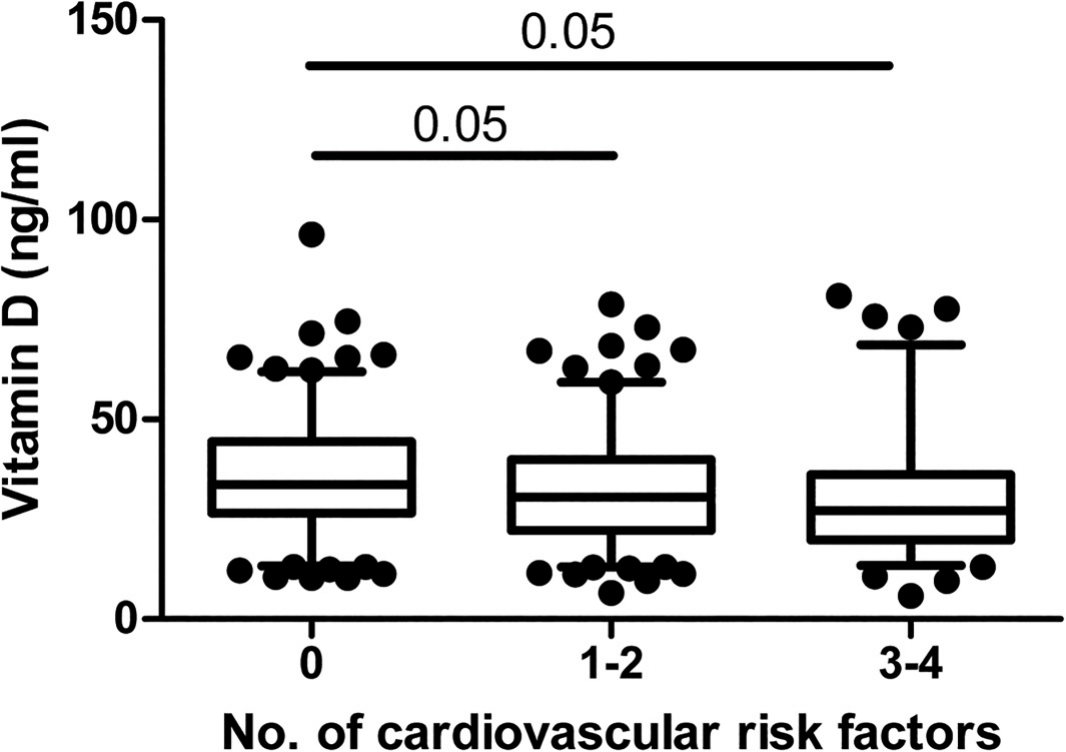
Plasma 25(OH)D_3_ concentration according to absence or presence of cardiometabolic risk factors. Central obesity: waist-to-height ration >0.5 [31]; elevated blood pressure (BP): systolic BP ≥130 mm Hg and/or diastolic BP ≥85 mm Hg; increased atherogenic risk: AIP≥0.11 [29]; and insulin resistance: QUICKI<0.322 were considered as factors indicated increased cardiometabolic risk. 0: no risk factor presented (n = 162); 1–2: subjects with 1 or 2 risk factors (n = 162); 3–4: subjects presenting 3 or 4 risk factors (n = 87). Data are given as interquartile range (box), 5^th^ and 95^th^ percentile (whiskers). Statistical evaluation was performed on natural ln-transformed data. ANOVA: p<0.001, Scheffe’s post hoc test p indicated.

25(OH)D_3_ levels ranged between 5.8–96.3 ng/ml (mean: 33.4 ± 14.5 ng/ml; Fig 1). 17.5% of subjects were 25(OH)D_3_ deficient, 28.7% presented 25(OH)D_3_ insufficiency, and satisfactory 25(OH)D_3_ levels were revealed in 53.8% of participants (Table 1).

### 25(OH)D_3_ levels and cardiometabolic risk factors

Cardiometabolic risk factors-free subjects presented significantly higher 25(OH)D_3_ levels (35.5 ± 14.2 ng/ml) in comparison with groups presenting increased (32.6 ± 14.3 ng/ml) and high (31.0± 15.1 ng/ml) number of cardiometabolic risk factors (Fig 1). However, the frequencies of 25(OH)D_3_ deficiency, insufficiency, and satisfactory levels did not differ significantly among the groups (Table 3). Mean 25(OH)D_3_ levels were similar among the groups presenting 25(OH)D_3_ deficiency, insufficiency, and satisfactory levels regardless of the presence or absence of cardiometabolic risk factors (Table 3). Gender or smoking status showed no significant impact on 25(OH)D_3_ concentrations either in the whole cohort, or in subgroups with regard to 25(OH)D_3_ status, and presence of cardiometabolic risk factors.

**Table 3:** 25(OH)D_3_ status and plasma levels according to presence of cardiometabolic risk factors.

		Number of cardiometabolic risk factors			
	All	0	1–2	3–4	p
**Frequencies (n; %)**					
Deficiency	72 (17.5%)	19 (11.7%)	31 (19.1%)	22 (25.3%)	0.053^Chi^
Insufficiency	118 (28.7%)	46 (28.4%)	45 (27.8%)	27 (31.0%)	
Satisfactory	221 (53.8%)	97 (59.1%)	86 (53.1%)	38 (43.7%)	
**Levels (ng/ml)**					
Deficiency	*15*.*5 ± 3*.*4*	*15*.*0 ± 3*.*3*	*15*.*4 ±3*.*3*	*16*.*0 ± 3*.*7*	*0*.*80*
Insufficiency	*26*.*3 ± 8*.*7*	*27*.*7 ± 10*.*7*	*24*.*6 ±2*.*7*	*27*.*0 ± 11*.*1*	*0*.*84*
Satisfactory	*43*.*1 ± 11*.*0*	*43*.*3 ± 10*.*3*	*43*.*0 ±11*.*3*	*42*.*5 ± 12*.*6*	*0*.*81*

Central obesity: waist-to-height ration >0.5 [31]; elevated BP: systolic BP (SBP)≥130 mm Hg and/or diastolic BP (DBP)≥85 mm Hg; increased atherogenic risk: AIP≥0.11 [29]; and insulin resistance: QUICKI<0.322 were considered as factors indicated increased cardiometabolic risk. 0: no risk factor presented (n = 162); 1–2: subjects with 1 or 2 risk factors (n = 162); 3–4: subjects presenting 3 or 4 risk factors (n = 87); chi: chi-square; otherwise ANOVA p indicated; *italics*: statistical evaluation performed on logarithmically transformed data

Thirty-eight % of subjects presented elevated BP values, 43% were centrally obese, 19% were insulin resistant, and 26% presented elevated atherogenicity of plasma. Those presenting elevated BP had significantly lower 25(OH)D_3_ levels in comparison with their normotensive counterparts (31.4 ± 14.5 ng/ml vs. 34.6 ± 14.4 ng/ml, p = 0.026). Presence of insulin resistance (31.2 ± 15.3 ng/ml vs. 33.9 ± 14.3 ng/ml, p = 0.053), central obesity (32.3 ± 14.7 ng/ml vs. 34.3 ± 14.3 ng/ml, p = 0.11), and increased atherogenic index of plasma (32.3 ± 14.7 ng/ml vs. 33.8 ± 14.4 ng/ml, p = 0.24) was reflected only with trends towards lower levels. In comparison with cardiometabolic risk factors-free subjects presenting mean 25(OH)D_3_ of 35.5 ± 14.2 ng/ml those suffering solely from insulin resistance displayed significantly lower concentrations (n = 12; 27.3 ± 13.1 ng/ml; p = 0.022), while in absence of other risk factors subjects presenting elevated BP (n = 40; 31.8 ± 15.6 ng/ml, p = 0.08); or central obesity (n = 31; 32.8 ± 14.0 ng/ml; p = 0.26), or increased atherogenic risk (n = 14; 30.4 ± 11.9 ng/ml; p = 0.13) showed only tendency towards lower 25(OH)D_3_ concentrations.

In Model 1 binary logistic regression indicated that in comparison with cardiometabolic risk factors-free subjects those presenting 1-to-2 cardiometabolic risk factors are 2.3-fold less likely to present satisfactory 25(OH)D_3_ status (B: -0.84, p = 0.015), and those presenting 3-to-4 risk factors are 4.3-fold less likely (B: -1.45, p = 0.001). Male gender (B: 0.48, p = 0.13) and ln eGFR (B: -1.35, p = 0.8) were not significant independent predictors. The model was significant (Omnibus test: 0.008), predicted correct classification in 76%, and the dependent variables accounted for 5-to7% of the variance (Cox & Snell and Nagelkarte square). Model 2 showed that elevated blood pressure and insulin resistance decrease the probability of being 25(OH)D_3_ sufficient (2.9-fold, p = 0.003 and 2.4-fold, p = 0.044, respectively); while the impact of gender, presence of central obesity, increased AIP and ln eGRF was insignificant (Omnibus test: 0.003, predicted correct classification: 76%, Cox & Snell and Nagelkarte square: 8% and 12%, respectively).

**Fig 2:**
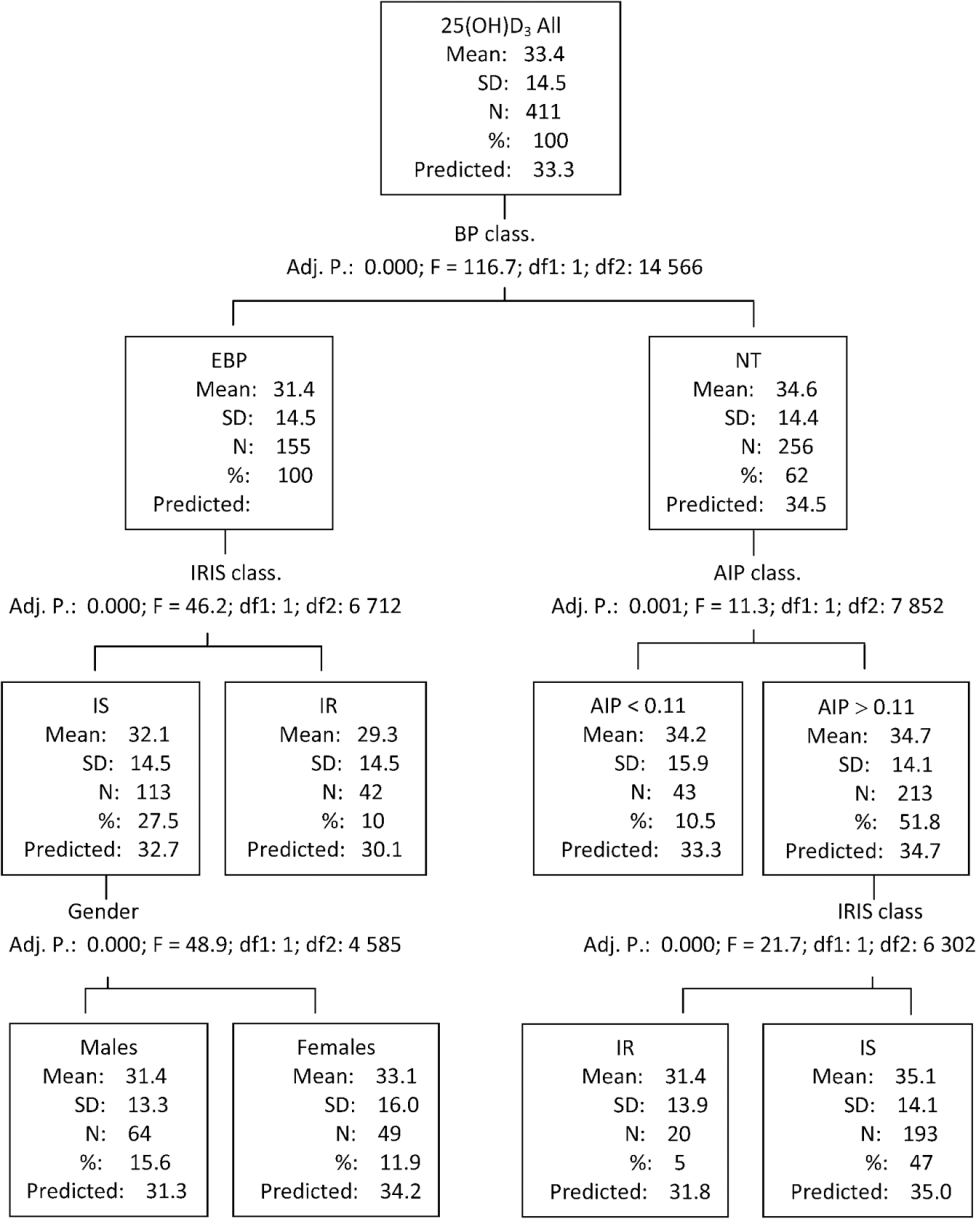
Decision tree. Std. Dev: standard deviation; BP: blood pressure; Adj.: adjusted; df: degree of freedom; EBP: elevated blood pressure (systolic BP ≥130 mm Hg and/or diastolic BP ≥85 mm Hg); NT: normotension; IRIS: insulin sensitivity (IS)/insulin resistance (IR); AIP: atherogenic index of plasma (AIP≥0.11 indicates increased atherogenic risk [29])

The impact of particular combination of CM risk factors on 25(OH)D_3_ levels was further investigated using SPSS AnswerTree, with age forced in as an influence variable (Fig 2). AnswerTree selected BP as a main determinant: Subjects presenting elevated BP displayed lower 25(OH)D_3_ levels in comparison with their normotensive counterparts. Insulin resistant subjects with elevated BP displayed the lowest 25(OH)D_3_ levels. All of them were also centrally obese (ICO: 0.62 ± 0.06), and all 27 subjects presenting 4 risk factors were assigned into this group. Among insulin sensitive subjects with elevated BP males presented lower 25(OH)D_3_ levels than females. Normotensives without increased atherogenic risk displayed slightly lower 25(OH)D_3_ levels if compared with their counterparts with increased atherogenic risk. However, in the former group insulin resistance was associated with the second lowest 25(OH)D_3_ levels. Forty percent of these subjects were centrally obese.

### Cardiometabolic risk factors and oxidative and inflammatory markers, adipokines

Due to shortage of plasma uric acid, sRAGE, CML, AOPPs and AGE-associated fluorescence could not be quantified in 54 subjects. With regard to the presence of cardiometabolic risk factors the groups differed significantly by all variables except for albuminemia, IL-6, hsTNF-α, and AOPPs/Alb levels (Table 2). If compared with cardiometabolic risk factors-free subjects those presenting 1 or 2 risk factors displayed significantly lower eGFR and adiponectin levels, higher uric acid and hsCRP concentrations, but similar levels of leptin, resistin, CML/Alb, AGE-Fl/Alb and sRAGE. Subjects bearing 3 and 4 risk factors displayed significantly lower eGFR, adiponectin, CML/Alb and sRAGE levels; and higher uric acid, hsCRP, leptin, resistin, and AGE-Fl/Alb levels in comparison with the risk factors-free subjects. Uric acid, hsCRP, leptin, resistin, and AGE-Fl/Alb levels were significantly higher and those of adiponectin, CML/Alb and sRAGE lower in subjects presenting high number of risk factors in comparison with their counterparts presenting 1 and 2 risk factors.

### Impact of 25(OH)D_3_ on oxidative and inflammatory markers, and adipokines

GLM with ln 25(OH)D_3_, ln eGFR, and cardiometabolic risk factors as covariates (or gender as a fixed factor if appropriate) did not reveal significant independent impact of 25(OH)D_3_ either on oxidative and inflammatory markers, or adipokines. Neither binary logistic model nor SPSS AnswerTree assigned either non-standard marker as independent determinant of 25(OH)D_3_ status.

## Discussion

To the best of our knowledge this is a first study examining the 25(OH)D_3_ status in relation to potential additive effect of manifested number of standard cardiometabolic risk factors in non-diabetic medication-free adults. Our data suggests, that in non-diabetic medication-free adults increasing number of cardiometabolic risk factors is associated with lower 25(OH)D_3_ levels. Elevated blood pressure combined with insulin resistance were associated with the lowest 25(OH)D_3_ levels; and low levels were also linked with insulin resistance in normotensive subjects not presenting increased atherogenic risk. It remains unclear whether hypovitaminosis D in apparently healthy medication free subjects manifests preferentially by increased BP and insulin resistance, or whether subjects presenting particular clustering of cardiometabolic risk factors are more prone to develop hypovitaminosis D. In the presence of cardiometabolic risk factors the levels of adipokines, biomarkers of inflammation, and oxidative stress were significantly altered. However, we did not reveal a significant impact of interaction between 25(OH)D_3_ levels and cardiometabolic risk factors on circulating non-standard biomarkers. This might imply that in apparently healthy non-diabetic adults different pathomechanisms than 25(OH)D_3_ deficiency *per se* play crucial role in the induction of microinflammation and oxidative stress, regulation of AGEs/RAGE axis, or modulation of adipokines.

### 25(OH)D_3_ status

In comparison to other Central European studies on general population in latitudes similar to that of Bratislava, our cohort presented mean 25(OH)D_3_ levels (33.4 ± 14.5 ng/ml) within the upper reported range and a rather high (almost 54%) prevalence of satisfactory 25(OH)D_3_ levels (>30 ng/ml) [32–34]. However, this finding might not accurately reflect 25(OH)D_3_ status in general population of Slovak adults, since participation in the study was on voluntary basis and it cannot be excluded that we investigated an “over-healthy” population. Data on vitamin D status in general Slovak population are not available. Small studies in different patients’ groups report 64%-to-100% prevalence of levels <30 ng/ml [35–38].

### 25(OH)D_3_ and standard cardiometabolic risk factors

#### Blood pressure and insulin resistance

In humans vitamin D deficiency associates with increased BP and the risk of developing hypertension (reviewed in [39, 40]). As expected, our subjects presenting cardiometabolic risk factors had elevated BP values (including MAP and PP) and showed lower 25(OH)D_3_ levels than their risk factors-free counterparts. Elevated BP appeared consistently as the most prominent cardiometabolic risk factor associated with low 25(OH)D_3_ levels. It remains unclear whether this observation reflects the high susceptibility of BP-regulating mechanisms to hypovitaminosis D *per se* or whether modulating factors (e.g. genetic, environmental, life-style, etc.) not followed in present study interplay.

Observational studies in humans reported positive association between vitamin D status and insulin secretion and insulin sensitivity (reviewed in [10, 41]). Recent meta-analyses of prospective studies in apparently healthy adults showed that having vitamin D levels in upper vs. lower tertile at baseline is associated with a 16% lower future risk of developing insulin resistance [42]. In our study, insulin resistant normotensive subjects not presenting increased atherogenic lipid profile displayed low 25(OH)D_3_ levels and the lowest mean 25(OH)D_3_ levels were revealed in insulin resistant subjects presenting elevated BP. The latter data match those of Abassi et al. showing that the most insulin resistant quartile of patients with essential hypertension presented the lowest 25(OH)D_3_ levels [43]. Interestingly, all our subjects assigned to the group with the lowest 25(OH)D_3_ levels were also centrally obese and 64% of them presented increased atherogenic risk. Whether particularly combination of elevated blood pressure and insulin resistance generally associates with lower 25(OH)D_3_ levels remains to be confirmed in further studies. If affirmed, it might be speculated that particularly hypertensive insulin resistant subjects would benefit from vitamin D repletion most.

#### Atherogenic dyslipidemia

Low 25(OH)D_3_ levels are associated with an unfavorable lipid profile [40, 44–47]. We confirmed the data from Canadian study, which showed that rising number of cardiometabolic risk factors is associated with worsening of lipid profile components as well as poorer vitamin D status [48]. However, our results do not support 25(OH)D_3_ status as a decisive factor in an induction of unfavorable lipid profile in apparently healthy general population; our normotensive subjects with increased atherogenic risk presented similar 25(OH)D_3_ levels if compared with their counterparts with low atherogenic risk. Atherogenic profile neither exhibited significant impact on 25(OH)D_3_ status in the binary logistic regression analysis, nor in the hypertensive branch of the decision tree. Thus, further studies in different populations are definitely needed to elucidate the association between vitamin D status and atherogenic risk with regard to the manifestation of other cardiometabolic risk factors.

#### Central obesity

Obese humans present generally low vitamin D status (reviewed in [49]). It remains unclear whether local conversion or trapping from circulation represent main source of 25(OH)D_3_ deposited in human adipose tissue [50]. Weight reduction is not associated with significant change in circulating 25(OH)D_3_ levels or its content in adipose tissue [50]. In our cohort central obesity did not appear as independent determinant of vitamin D status either *per se* or in combination with any other risk factor, even though lean to markedly centrally obese (ICO > 0.6) subjects were included. Life-style associated factors not tracked in our study, such as physical activity particularly if performed outdoors, dietary intake of vitamin D, or genetic variations, and duration of obesity could play a role.

#### 25(OH)D_3_ and non-standard cardiometabolic risk factors

Several lines of evidence point to strong associations between oxidative stress, microinflammation, and cardiovascular risk. Inflammation and oxidative stress act as cooperative and synergistic partners in the pathogenesis of hypertension (reviewed in [21]). Obesity is closely linked to oxidative stress and low-grade systemic inflammation with subsequent dysregulation of adipokines and inflammatory cytokine production [51]. Insulin resistance may be at least partially mediated by mechanism involving oxidative stress (implying particularly angiotensin II) [52], and low grade inflammation [53]. Both, inflammatory mechanisms and enhanced lipid oxidation may lead from dyslipidemia to atherogenesis [54, 55]. The inflammatory response, production of reactive oxygen species and adipokines, among others, are affected by vitamin D [1, 8, 9, 11, 12].

#### Inflammatory and oxidative markers

Elevated levels of CRP, IL-6, TNF-α or AOPPs (markers of phagocyte-mediated oxidative stress and inflammatory syndrome) predict future cardiovascular events or increased risk of T2DM in apparently healthy subjects or non-diabetic patients with decreased renal function [56–59]. In different populations (i.e. hypertensives, obese subjects, elderly), 25(OH)D_3_ levels associate inversely with multiple inflammatory markers (i.e. hsCRP, hsTNF-α, AOPPs, pro-inflammatory cytokines), suggesting a potential anti-inflammatory role for vitamin D [20, 60–62]. In our study increasing number of cardiometabolic risk factors was associated with higher hsCRP, while levels of IL-6, hsTNF-α and AOPP/Alb were similar. IL-6 and hsTNF-α showed no significant association with 25(OH)D_3_ levels, while those of hsCRP and AOPP/Alb could not be reliably approximated due to non-linear distribution of these markers even after their log transformation. Thus, the clinical impact of anti-inflammatory action of vitamin D in general population remains uncertain.

#### Advanced glycation end products and sRAGE

AGEs, formed by non-enzymatic glycation of proteins and under enhanced oxidative and carbonyl stress, exert inflammatory, pro-oxidant, and atherogenic effects via interaction with specific cell-surface receptor–RAGE [63]. Circulating sRAGE may act as a decoy [63]. Rise in circulating AGE and decline in sRAGE levels accompany hypertension, obesity, cardiovascular, and renal diseases; while both AGEs and sRAGE are elevated in diabetes [64–67].

In our study presence of ≥ 3 cardiometabolic risk factors was associated with the highest AGE-Fl/Alb, while with the lowest CML/Alb levels. AGE-Fl/Alb represent a bulk estimate of circulating AGE products with intrinsic fluorescence, while CML is a non-fluorescent and the most abundant circulating AGE compound, acting as a RAGE ligand [27]. Obesity-, elevated blood pressure-, insulin resistance- or atherogenic lipid profile-associated enhanced oxidative stress might trigger AGE formation. The apparently contradictory finding of lower CML/Alb in subjects presenting cardiometabolic risk factors probably reflects trapping of lipophilic CML into fat tissue, resulting in low levels of circulating CML in obese subjects [68, 69]. We did not reveal significant association between AGE/Alb or CML/Alb levels and the 25(OH)D_3_. However, in streptozotocin-induced diabetic rats increased deposition of CML in aortic wall was blunted by administration of cholecalciferol, affecting probably the oxidative-stress mediated pathways [70]. To the best of our knowledge no human data on the effect of vitamin D supplementation on circulating or tissue AGEs are currently available.

sRAGE levels were decreased in subjects presenting ≤3 cardiometabolic risk factors, but no significant association between sRAGE and the 25(OH)D_3_ status was revealed. Experimental and clinical data suggest that vitamin D might affect RAGE. In cell culture studies calcitriol blunted the AGEs- or lipopolysaccharide-induced up-regulation of RAGE mRNA and protein and counteracted their stimulating effect on NF-κB pathway [71, 72]. In streptozotocin-induced diabetic rats calcitriol treatment attenuated the increased expressions of cardiac RAGE, probably via modulating effect on angiotensin II receptor 1 [73]. Administration of calcitriol to 25(OH)D_3_ deficient women with polycystic ovary syndrome was associated with rise in circulating sRAGE and an increase in sRAGE positively correlated with that of serum 25(OH)D_3_ [74]. The mechanisms are unclear, but active vitamin D enhances the expression of matrix metalloproteinase-9 (MMP-9) shedding the cell-surface located RAGE [75, 76]. Thus, elucidation of potential functional relationship between the 25(OH)D_3_ status and RAGE or sRAGE in apparent health requires further studies.

#### Adipokines

Large population studies indicate a direct relationship between serum 25(OH)D_3_ and adiponectin [77, 78]. A smaller study reported a negative correlation with leptin, no relationship to adiponectin and resistin in healthy population, and no significant relationship in morbidly obese subjects [79]. In our study adipokine levels were significantly altered in the presence of cardiometabolic risk factors, but no association with the 25(OH)D_3_ status was revealed. Reasons for discordance in clinical data remain unclear.

### Limitations

The strength of our study is the large number of investigated non-diabetic medication-free adults not supplemented with 25(OH)D_3_, recruited from restricted geographical area of Western Slovakia; and a wide scale of investigated non-standard cardiometabolic risk factors. However, the study encounters several limitations. 25(OH)D_3_ status determination and the classification of presence of cardiometabolic risk factors was based on a single examination. We recruited approximately 50% less subjects presenting 3-to-4 cardiometabolic risk factors in comparison with other 2 groups, mirroring the fact that generally such subjects are on medication. Our subjects were not completely independent—family and household members participated. Nutritional intake of vitamin D was not ascertained. Being cross-sectional in its nature, our study allows commenting only on associations–pathomechanisms involved, causality or the direction of potential causal associations cannot be determined. Additional limitations are discussed above in relation to pertinent subject.

## Conclusion

In non-diabetic medication-free adults specific combination of manifested cardiometabolic risk factors rather than their number associates with lower 25(OH)D_3_ levels. Elucidation of hypovitaminosis D association with particular combinations of classical cardiometabolic risk factors might be of clinical importance in identification of the subjects who are most likely to benefit from vitamin D supplementation. The modulatory impact of 25(OH)D_3_ on classical as well as non-standard cardiometabolic risk factors (e.i. inflammatory and oxidative stress markers, adipokines and AGE/RAGE axis) definitely requires further studies, as it might be disparate in apparent health and under disease-imposed burden.
